# Severe community-acquired pneumonia caused by *Chlamydia psittaci* genotype E/B strain circulating among geese in Lishui city, Zhejiang province, China

**DOI:** 10.1080/22221751.2022.2140606

**Published:** 2022-11-10

**Authors:** Xin-Cheng Qin, Jinwei Huang, Zhangnv Yang, Xiangrong Sun, Wen Wang, Enhui Gong, Zhuo Cao, Jianfeng Lin, Yanai Qiu, Bohai Wen, Biao Kan, Jianguo Xu, Tian Qin

**Affiliations:** a State Key Laboratory of Infectious Disease Prevention and Control, Collaborative Innovation Center for Diagnosis and Treatment of Infectious Diseases, National Institute for Communicable Disease Control and Prevention, Chinese Center for Disease Control and Prevention, Beijing, People’s Republic of China; b Department of Respiratory and Critical Care Medicine, The Sixth Affiliated Hospital of Wenzhou Medical University, Lishui, People’s Republic of China; c Key Laboratory of Vaccine, Prevention and Control of Infectious Disease of Zhejiang Province, Zhejiang Provincial Center for Disease Control and Prevention, Hangzhou, People’s Republic of China; d Department of Clinical Laboratory, Nanchang Center for Disease Control and Prevention, Nanchang, People’s Republic of China; e Department of Internal Medicine, Suichang Hospital of traditional Chinese medicine, Lishui, People’s Republic of China; f Pathogen and Biosecurity, Beijing Institute of Microbiology and Epidemiology, Beijing, People’s Republic of China

**Keywords:** Community-acquired pneumonia, psittacosis, *Chlamydia psittaci*, poultry, zoonotic potential

## Abstract

Between November 2021 and January 2022, four patients of community-acquired pneumonia were admitted to the hospitals in Lishui city, Zhejiang province, China. Their main clinical manifestations were fever and dry cough as well as radiographic infiltrate, but the empiric antimicrobial therapy or traditional Chinese medicine was not effective for their illness. Clinical specimens from the patients as well as environmental and poultry specimens were collected for the determination of the causative pathogen. The *ompA* gene and seven housekeeping genes of *Chlamydia psittaci* were successfully amplified from all the patients, and the sequences of each gene were identical to one another, suggesting that they were infected by the same strain of *C. psittaci*. A novel strain of *C. psittaci* (LS strain) was isolated from the bronchoalveolar lavage fluid of patient 2 and its whole genome was obtained. Phylogenetic analyses based on the whole-genome sequences showed that the isolate is most closely related to the strain (WS/RT/E30) identified as genotype E/B. In addition, The *ompA* gene and four housekeeping genes of *C. psittaci* were also amplified from two of four faeces samples of geese at the home of patient 2, and the sequences from geese were 100% identical to those from the patients. Accordingly, these cases could be attributed to a circulating *C. psittaci* strain of genotype E/B in the local geese. Therefore, there is an urgent need to strengthen the regional surveillance on *C. psittaci* among poultry and humans for prevention and control of the outbreak of psittacosis in the city.

## Introduction

From November 2021 to January 2022, four consecutive cases of severe community-acquired pneumonia (CAP) were admitted to the hospitals in Lishui city, Zhejiang province, China. Their main clinical manifestations were fever, dry cough, and lung infiltrate, but the empiric antimicrobial therapy or traditional Chinese medicine was not effective for their illness. Therefore, the aetiological and epidemiological investigation of the CAP in Lishui city was initiated.

Community-acquired pneumonia can be caused by viruses, bacteria, or fungi. In bacterial pathogens, *Streptococcus pneumoniae* (frequency, 14.0%), *Staphylococcus aureus* (5.1%), *Mycoplasma pneumoniae* (1.9%), *Legionella pneumophila* (1.4%), and *Escherichia coli* (1.4%) commonly cause CAP, whereas *Chlamydophila pneumoniae*, *Pseudomonas* spp., *Haemophilus influenzae*, and *Mycobacterium tuberculosis* are less common (<1%), and *Chlamydia psittaci* is far less common such as exposure to birds/poultry [1]. The patients infected with *C. psittaci* do not respond to usual therapy. A prospective population-based surveillance study conducted at three large hospitals in the USA found that despite comprehensive diagnostic testing, a causative organism was isolated in only 38% of CAP cases [2].

Here, we present four consecutive cases of *C. psittaci* pneumonia in Lishui city, and show how the etiologic diagnosis was facilitated by the use of molecular methods and isolation of the infective agent. Through the recent study, we found that these human cases of psittacosis probably originated from the geese population infected with *C. psittaci* in Lishui city. The results highlight the importance of strengthening the surveillance of *C. psittaci* to avoid outbreaks of psittacosis in the city.

## Materials and methods

### Sample collection

Four patients were admitted to the hospitals because of chills and fever accompanied by dry cough for 3–10 days from 13 November 2021 to 9 January 2022. None of the patients responded to the empirical antimicrobial therapy or traditional Chinese medicine. The respiratory tract specimens including throat swab, sputum, and bronchoalveolar lavage fluid (BAFL) were collected from the patients when available. Serum samples from patients and their family were collected seven to nine months later for retrospective serological analysis.

The clinical data of the patients were collected. The clinical examination indexes such as white blood cell count, neutrophil percentage, and C-reactive protein (CRP) level, as well as comprehensive computed tomography (CT) data were obtained. Unless otherwise specified, the indexes were measured on the day of admission or transfer to the hospital. The data about antibiotics treatment and outcomes as well as any relevant follow-up findings were also recorded. After confirming *C. psittaci* infection, the environmental and poultry samples at the homes of patients were also collected if available. Serum samples were collected from the patients and their family members who live together when available.

All patients provided written informed consent, and the research protocol was reviewed and approved by the ethics committees of the National institute of communicable disease prevention and control, China CDC (ICDC-2019012).

### Polymerase chain reaction assays and sequencing

DNA was extracted from the patients’ respiratory tract specimens and the poultry samples as well as the environmental samples using QIAamp DNA Mini Kit (QIAGEN, Hilden, Germany) according to the manufacturer’s instructions. The extracted DNA samples were detected using polymerase chain reaction (PCR) targeting the *ompA* of *C. psittaci*. We also attempted to amplify seven housekeeping genes of *C. psittaci* [3]. The amplified fragments in PCR were purified and sequenced by the company (Sangon, Shanghai, China).

### Pathogen isolation

The BALF specimen from patient 2 was used to inoculate L929 cells, trying to isolate the suspected pathogen according to previously published protocols [4]. The cells were cultivated in Dulbecco’s modified Eagle’s medium with 2% foetal bovine serum at 37°C in 5% carbon dioxide, supplemented with vancomycin and amphotericin. The proliferation of *C. psittaci* was detected by comparing the content of *C. psittaci* between the cultures and the original samples using quantitative PCR [5]. After confirming the successful isolation of *C. psittaci*, the isolate was inoculated into the L929 cells cultured in vitro, and then the cells were examined for intracellular chlamydial inclusions under a light microscope after Wright-Giemsa staining three days after inoculation.

### Transmission electron microscopy

The L929 cells were collected three days after inoculation, and the cells were then fixed with 2% paraformaldehyde – 2.5% glutaraldehyde and were then incubated with 1% osmium tetroxide. Samples were dehydrated in a graded ethanol series and embedded in PON812 resin for sectioning [6]. Sections were stained with uranyl acetate and lead citrate, separately. The ultrathin sections were observed under a transmission electron microscope.

### mNGS sequencing, genome assembly, and core-gene analysis

DNA was quantified in a Qubit fluorometer. The complete *C. psittaci* genome was sequenced using both Illumina Hiseq and MinION Nanopore platforms, and the reads from both platforms were combined at multiple levels in order to obtain a reliable assembly. The process was described in detail in the supplementary materials.

### Serological assay

The serum-specific IgM and IgG antibodies against *C. Psittaci* were detected in serum samples using commercial IFA kits (Savyon Diagnostics Ltd., Ashdod, Israel) following the manufacturer’s instructions. We detected the IgM and IgG serum endpoint titres by serial 2-fold dilution. A titre of 1:20 or higher was considered as IgM positive and a titre of 1:64 or higher was considered as IgG positive.

## Results

### Case presentation

#### Case 1

A 48-year-old man presented with a sudden onset of fever, with a high temperature of 41°C. His other symptoms included chill, headaches, fatigue, and a dry throat. When he was first hospitalized at the local hospital, the chest computed tomography (CT) scan showed bilateral lesion, lobar pneumonia in the upper lobe of the right lung, and pleural effusion on the right side. The patient was received an intravenous route anti-infection treatment with cefoperazone sulbactam sodium (3 g ivgtt q8h) combined with levofloxacin (0.4 g ivgtt qd). Three days after illness onset, the patient developed a paroxysmal cough with a little sticky white sputum. On day 6 after illness onset, the patient was transferred to the large urban hospital for further treatment. At the hospital, he was diagnosed with severe pneumonia and respiratory failure. The patient was received treatments including meropenem (1.0 g ivgtt q8h) for anti-infection, ambroxol (30 mg ivgtt bid) for sputum reduction and other symptomatic supportive treatment. As a result, his clinical sign decreased but the fever persisted. The BALF sample of the patient was collected and the *ompA* gene of *C. psittaci* was amplified from the sample by PCR on day 9 after admission. And then he was administrated with doxycycline (100 mg po q12h), and his fever subsided and his symptoms were gradually improved after taking it.

#### Case 2

**Figure 1.**
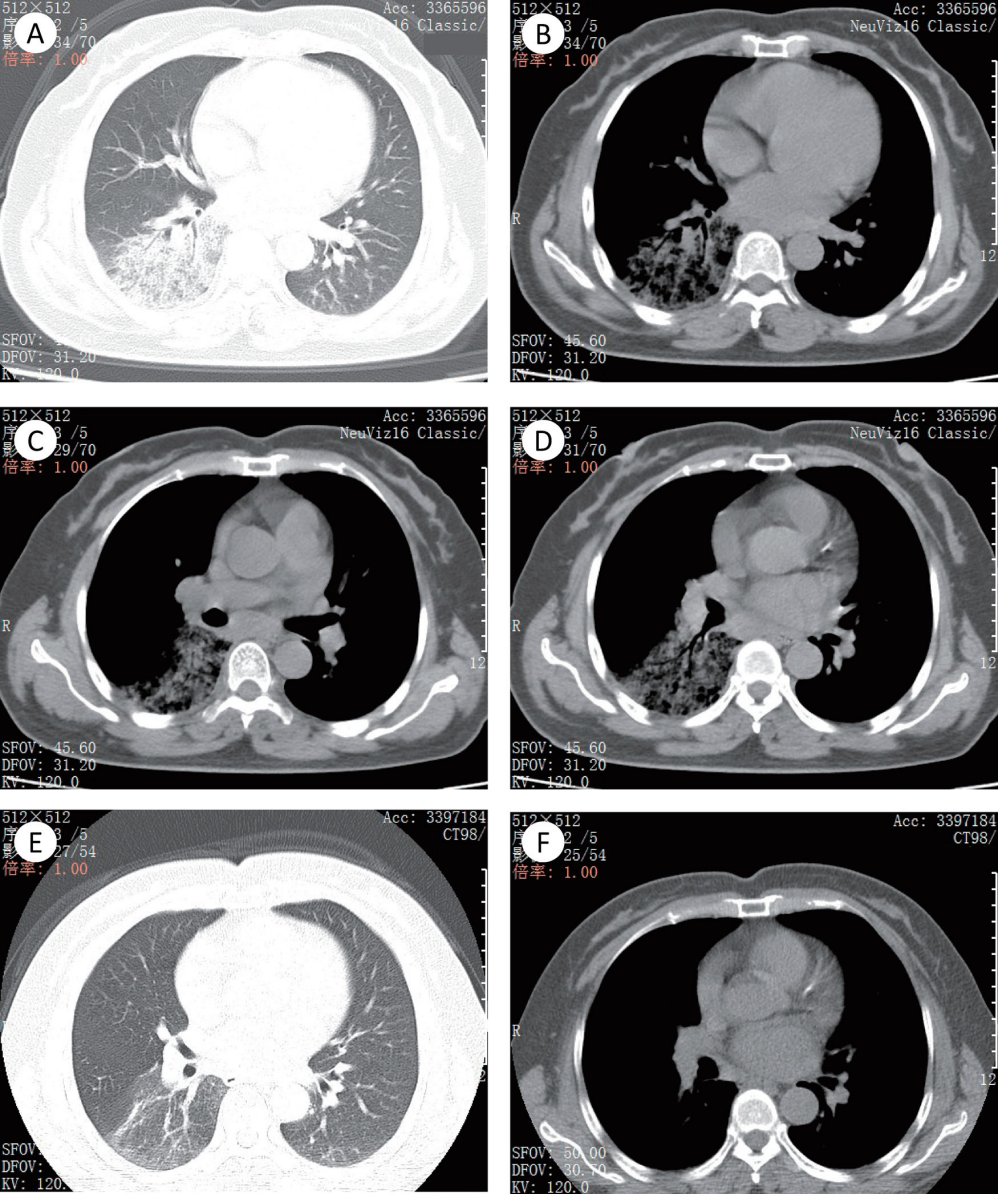
The imaging findings of patient 2. (A)–(D) The pulmonary window (A) and mediastinal window (B) showed the right lower lobe with segmentally distributed ground-glass opacity (GGO), with slightly uneven density and fuzzy boundary. Air bronchogram is presented in the lesion parenchyma. (C) and (D) Enlarged lymph node could be seen in mediastinal lymph nodes station 7 and 8. (E) and (F) The pulmonary window and mediastinal window showed that the pulmonary lesions were absorbed obviously, with a little light ground glass shadow left. Mediastinal lymph nodes were also significantly absorbed and shrunk.

A 56-year-old woman presented with cough accompanied by little yellow phlegm. She had a fever, with a high body temperature of 38.3°C. She experienced no chest pain or haemoptysis. She was diagnosed as having upper respiratory tract infection in the outpatient service of the hospital. However, she showed no significant improvement after a short course therapy with cefuroxime (0.5 g bid) combined with levofloxacin (0.4 g qd) orally for five days. She was admitted to the large hospital 10 days after the onset of illness. After admission, the chest CT scan suggested inflammation in the right lower lobe (Figure 1(a,b)) and shown swollen lymph nodes (Figure 1(c,d)). The piperacillin-tazobactam (4.5 g ivgtt q8h) was administrated for anti-infection, ambroxol (30mg ivgtt bid) for reduction of sputum viscosity, and glutathione (1.2 g ivgtt qd) for liver protection. Three days after these treatments, the body temperature did not subside significantly. And then her BALF sample was collected for PCR targeting to the *ompA* of *C. psittaci*. After the positive PCR result, she was administered with doxycycline (100 mg po q12h). Her fever subsided and clinical symptoms were relieved, as well as pneumonia was improved in radiological findings (Figure 1(e,f)) by treatment with doxycycline.

#### Case 3

A 54-year-old woman developed fever and chills, accompanied by paroxysmal dry cough. She experienced no improvement after self-medication with Chinese herbal medicine. After admission to the hospital, the chest CT scan suggested that inflammation in the left upper lobe. The piperacillin-tazobactam (4.5 g ivgtt q8h) therapy was given for anti-infection, and compound methoxyphenamine capsules (two tablets po tid) for relieving cough. However, the patient’s cough became worsen after three days of anti-infection treatment with piperacillin-tazobactam. She felt slightly shortness of breath, the fever did not subside, and the CRP level elevated slightly. By enquiring the poultry exposure history, the patient had a history of butchering geese a week before the onset of illness. Therefore, her throat swab sample was collected for PCR assay targeting *ompA* of *C. psittaci*. After the positive PCR result, she was administered with doxycycline (100 mg po q12h). After taking it, her fever subsided and clinical signs were improved.

#### Case 4

A 54-years old man experienced dizziness and fatigue without an underlying cause at the illness onset. He was running a low-grade fever (unmeasured temperature) without any other sign of infection. On day 3 after fever onset, he was admitted to the hospital and diagnosed as having CAP. His sputum sample was positively detected by PCR targeting the *ompA* of *C. psittaci*. After taking doxycycline (100 mg po q12h), his fever subsided.

Patients 1–3 underwent chest CT scanning. Lesions were more likely to be unilateral (Table 1), whereas pleural effusion was found in the severe case. All the patients had markedly elevated levels of the serum C-reactive protein (CRP), and all the patients (except patient 3) had elevated levels of neutrophils per cent. Patient 1 had elevated levels in both white-cell count and neutrophils per cent (Table 1).

**Table 1.** Clinical characterization and laboratory findings of the patients.

	Patient 1	Patient 2	Patient 3	Patient 4
*Clinical characterization*				
Temperature (°C)^#^	41	38.3	38.1	37.7
Asthenia	Y	N	Y	Y
Anorexia	N	Y	N	N
Myalgia	N	N	Y	N
Chill	Y	Y	Y	Y
Headache	Y	N	N	N
Pharyngoxerosis	Y	N	N	N
Pharynx ache	N	N	N	N
Dry cough	Y	Y	Y	N
Dizziness	N	N	N	Y
Laboratory findings*				
White blood cell count	14.1 × 10^9^/L	7.7 × 10^9^/L	7.9 × 10^9^/L	8.1 × 10^9^/L
Neutrophils per cent	88.70%	84.90%	69.80%	86.20%
CRP (mg/L)	191.3	261.1	106.2	113.5
*Image findings*				
Chest CT	Bilateral inflammation, lobar pneumonia in the upper lobe of right lung	Inflammation in the lower lobe of right lung	Inflammation in the upper lobe of left lung	NA

Y, yes; N, no; NA, non-available.

^#^ Temperature measured on the day of admission or transfer to the hospital.

*Normal ranges: white-cell count: 3.5−9.5 × 10^9^/L, Neutrophil per cent: 50–70%, CRP: 0–10 mg/L.

### Etiological and epidemiological investigation

A 1206 bp fragment of *ompA* of *C. psittaci* was amplified from the patient’s samples. The nucleotide sequences of the *ompA* fragments amplified from the patients were identical to one another, which shared 100% nucleotide similarities to that of the strain SZ15 (MK630234) isolated from laying ducks in Guangdong province, China [7].

During the questionnaire survey, we learned that patient 2 raised ducks and geese at home, patient 3 had the history of butchering geese one week before the illness onset, the other two patients denied having any history of close contact with live poultry. Sixty-six samples, including chickens faeces, ducks faeces, geese faeces, and environmental samples, were collected at the poultry houses of patient 2 (Table S1). In PCR assay, the *ompA* fragments of *C. psittaci* were positively amplified from two out of four geese faeces samples, which were identical to those amplified from the patients in DNA sequence analysis. All of the seven housekeeping genes (*enoA*, *fumC*, *gatA*, *gidA*, *hemN*, *hlfX*, and *oppA*) were amplified from the four patients using the primers previously described. But only four genes (*enoA*, *gatA*, *hemN*, and *hlfX*) of the seven genes were amplified from the geese faeces. The four genes from geese were identical to those from the patients in DNA sequence comparison.

**Figure 2.**
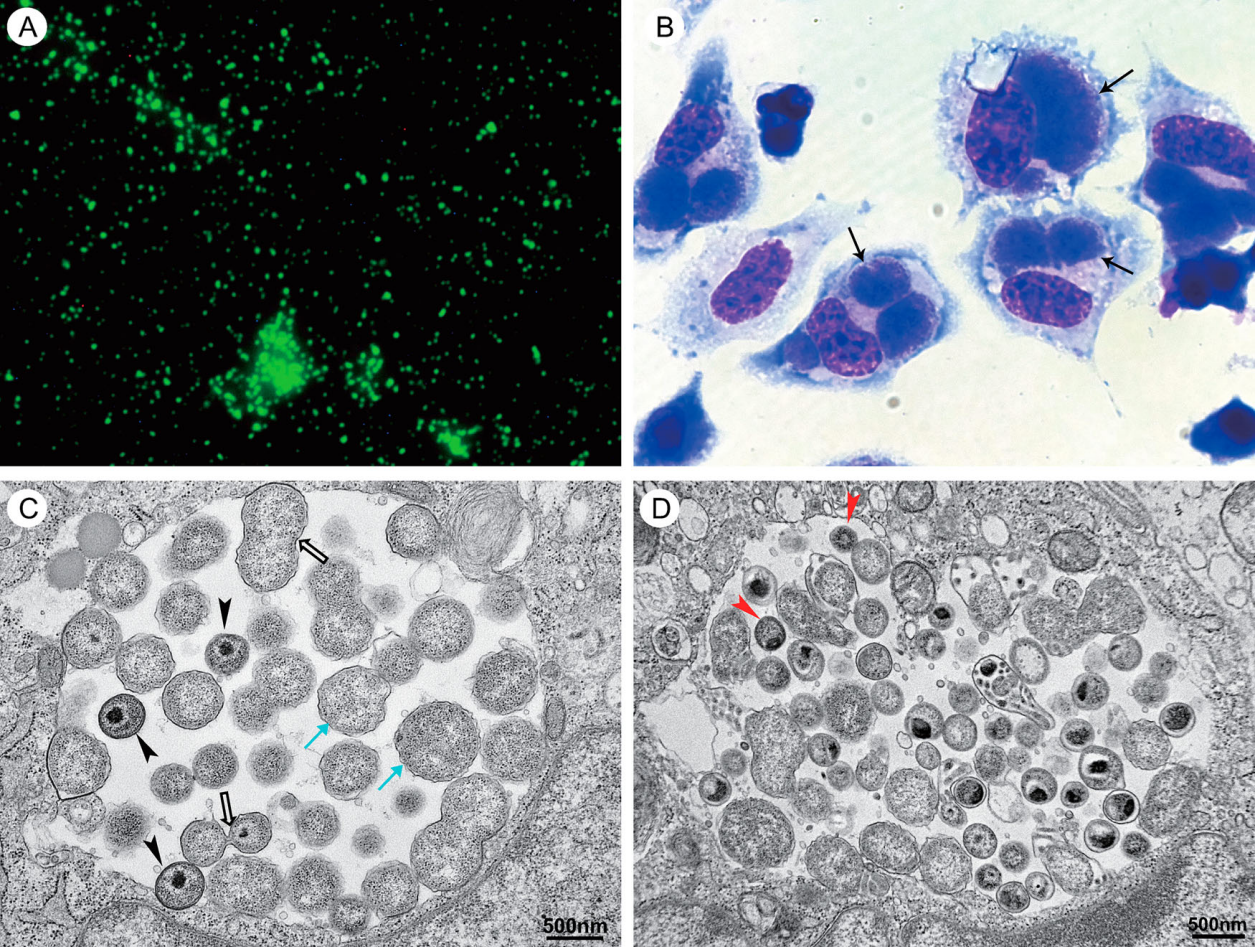
Photomicrographs of cells infected with *C. psittaci* LS strain. (A) Immunofluorescent staining with one patient’ serum under immunofluorescent microscopy at 400× magnification (Eclipse 80i, Nikon, Shanghai, China). (B) Wright-Giemsa staining under light microscopy at 1000× magnification (Eclipse 80i, Nikon, Shanghai, China). The inclusions of *C. psittaci* are indicated by black arrows. (C) The different morphological forms of *C. psittaci* present within a single inclusion in the infected cell under electron microscopy (FEI Tecnai12, Thermo Fisher, Waltham, MA). The cyan arrows indicated the reticulate bodies and the hollow arrows indicated the dividing reticulate bodies. The arrowhead indicated the intermediate bodies. (D) The red arrows indicated the elementary bodies under electron microscopy.

Serum samples were collected from four patients and four family members for retrospective serological analysis. All the patients’ samples were positive for *C. psittaci* IgG antibodies (titre higher than or equal to 1:64). Three family members were positive, and one had a titre as high as 1:512 (Table S2, Figure 2(a)). Samples from both the patients and family were negative for *C. psittaci* IgM antibodies.

### Isolation of *C. Psittaci* and genome analysis

*Chlamydia psittaci* was successfully isolated from the BALF samples from patient 2 using L929 cell culture. Changes in cell morphology included rounding, detaching, and cell debris as a result of cell death were noted 12 days after inoculation. The detaching cells were collected to inoculate into L929 cells cultured in vitro. Three days after inoculation, the cells were collected. Giemsa staining revealed the big inclusion of *C. psittaci* in the cytoplasm of L929 cells under light microscopy (Figure 2(b)), which are designated as LS strain. The different morphological forms of *C. psittaci* in the infected cells were observed under electron microscopy (Figure 2(c,d)).

**Figure 3.**
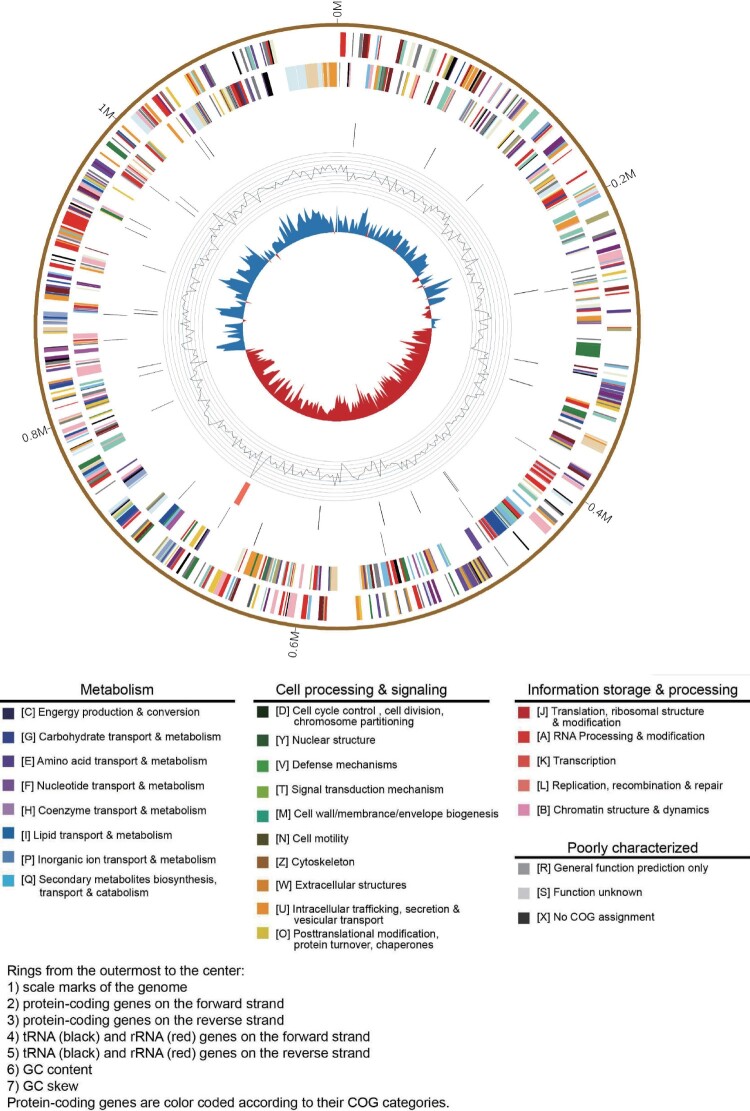
Genome features of *C. psittaci* LS strain. Circular representation of the *C. psittaci* LS strain genome.

**Figure 4.**
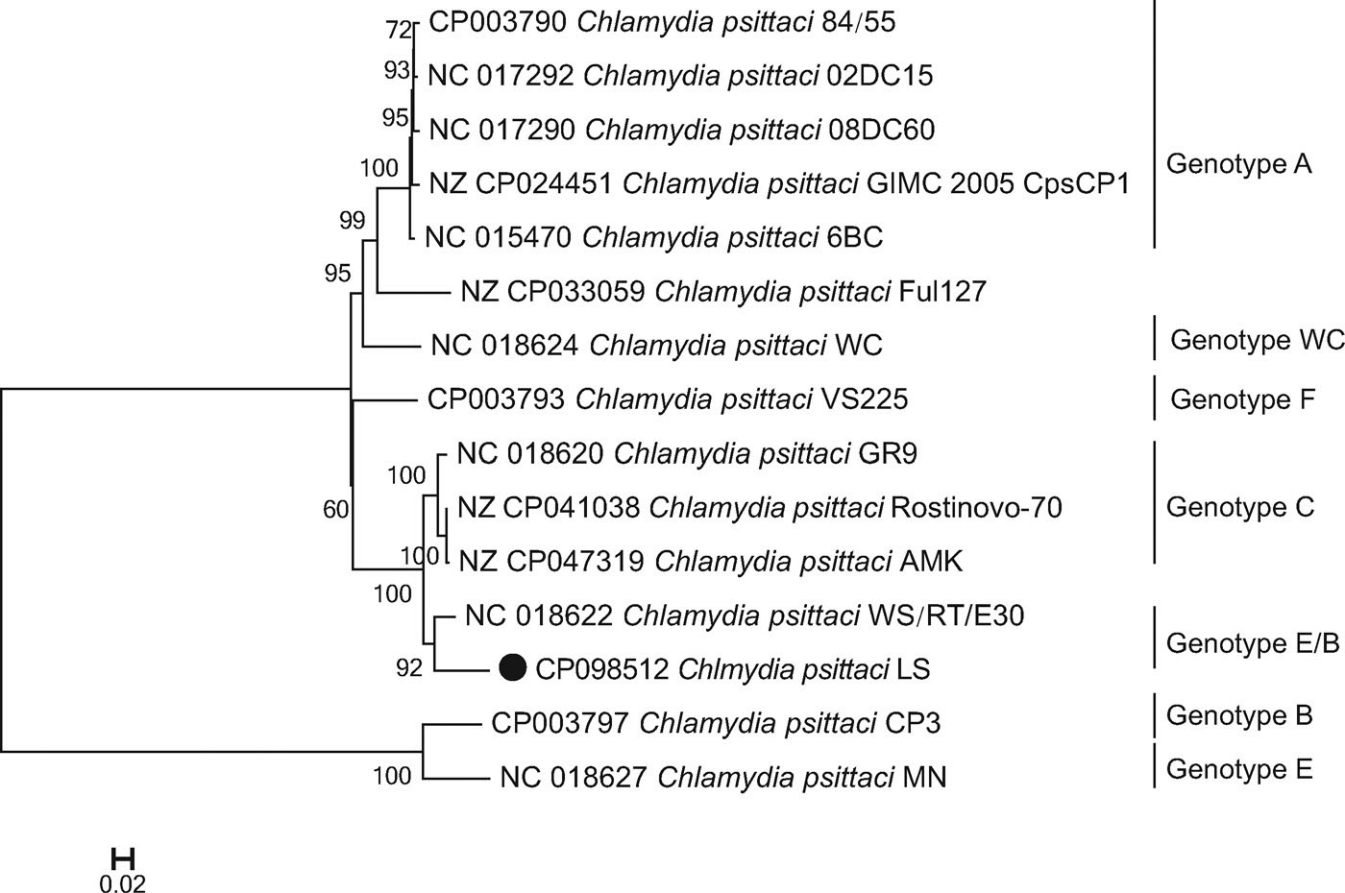
Phylogenetic relationship of *C. psittaci* LS strain and other genotypes of *C. psittaci.* Maximum-likelihood phylogeny for *Chlamydia*. The phylogeny was based on the alignment of concatenated core genes with the recombination region removed. The GenBank accession number of the whole-genome sequence of LS strain isolated in the present study is CP098512.

The whole-genome sequence of *C. psittaci* LS strain was obtained from the infected L929 cells. The genome size was 1,146,774 bp, with an overall GC content of 39.01%. Like most of the *C. psittaci* strains, it contains a 7702 bp plasmid. The features of LS strain are summarized in Table S3. The genome comparison revealed that *C. Psittaci* LS strain shared 935 core genes with representative strain of A–F and E/B genotypes, and 59 genes were unique to LS strain (Table S3, Figure S1). As visualized in Figure 3, the whole genome of LS strain included 1094 CDS. The main components of the core genome were associated with metabolism, cell processing, and signalling, as well as information storage and processing. We analysed LS genome compared with 14 *C. psittaci* genomes from diverse strains (Table S4) representing different known genotypes of the *C. psittaci*, which can infect a range of birds/poultry and mammals as well as humans. The maximum-likelihood tree (Figure 4) was constructed based on single nucleotide polymorphisms (SNPs) of the genome sequences, strongly suggesting that the *C. psittaci* LS strain isolated in the present study is most closely related to WS/RT/E30 strain that was isolated from *Anas platyrhynchos* in German and identified belongs to genotype E/B [8].

## Discussion

From November 2021 to January 2022, four consecutive cases of CAP were admitted to the hospitals in Lishui city, Zhejiang province, China. They presented with fever, dry cough, and pulmonary infiltration. All the patients except patient 4 were empirically treated with antimicrobial therapy following Chinese “Guidelines for the diagnosis and treatment of community-acquired pneumonia,” but the therapy was not effective for their illness, suggesting the CAP was caused by a specific pathogen such as *C. psittaci*. Therefore, the respiratory samples from the patients were detected by PCR targeting *ompA* of *C. psittaci* and the BALF samples from the patients were used to inoculate L929 cells to isolate causing pathogen from the patients. Finally, the chlamydial inclusions were observed in the cytoplasm of L929 cells inoculated with BALF from patient 2 under light microscopy, while electron microscopy showed that the different forms of chlamydial organisms present in the single inclusion, indicating that *Chlamydia* sp. was successfully isolated from patient 2. The whole-genomic sequence of this isolate was obtained from the host cells by mNGS method, which demonstrated that the isolate was a novel strain of *C. psittaci* and named as LS.

Whole-genome sequencing and subsequent comparative genomic analysis have become standard in analysis of the biology, virulence factors, evolution, and phylogenetic relationships of chlamydial organisms [9]. The whole-genomic sequence of LS strain was used in phylogenetic analysis, compared with the whole-genomic sequences of the known diverse strains of *C. psittaci*, representing the nine known genotypes (A to F, E/B, WC, and M56) of the *C. psittaci*, which can infect a range of birds/poultry and mammals as well as humans. *C. psittaci* are currently divided into 15 genotypes by using DNA microarray assay based on sequences of *C. psittaci ompA*: A–F, E/B, WC, M56, 1V, 6N, Mat116, R54, YP54, and CPX0308 [10]. Genotypes A–F and E/B are clustered in a certain host preference. Genotypes A and B are associated with *Psittaciformes* birds and *Columbiformes* birds. Genotype C has been isolated from poultry (ducks and geese), whereas genotype D has been found mainly in turkeys. The host range of genotype E is more diverse, since it has been isolated from pigeons, ratites, ducks, turkeys, and occasionally humans. Genotype F has been isolated from birds and turkeys. Genotypes WC and M56 are associated with cattle and muskrats, respectively [11,12]. Genotype E/B has been isolated mainly from ducks [13].

The maximum-likelihood tree (Figure 1) based on single nucleotide polymorphisms (SNPs) shown that LS strain was most closely related to WS/RT/E30 strain that was isolated from *A. platyrhynchos* in German and identified belongs to genotype E/B. Therefore, it is strongly suggested that the *C. psittaci* LS strain belongs to *C. psittaci* genotype E/B that was mostly found in ducks and might be transmitted from parrots to humans [14].

Meanwhile, *C. psittaci ompA* and the seven housekeeping gene fragments were amplified from the samples from the four patients in PCR assay and they were identical to one another in DNA sequence analysis, which strongly indicates that the four patients suffered with CAP were all infected by *C. psittaci* genotype E/B strain*.* The four patients in our study presented with clinical manifestations ranging from mild to severe pneumonia, suggesting that the genotype E/B strains of *C. psittaci* might be highly virulent. Meanwhile, it was found that these patients responded well to treatment with tetracycline, demonstrating genotype E/B strain of *C. psittaci* was sensitive to the antibacterial antibiotics.

In addition, the environmental and poultry specimens from the home of patient 2 were also collected to detect the *C. psittaci ompA* in PCR assay. As a result, two of four faeces samples of the geese were positively detected by the PCR and the DNA sequences of the *ompA*, *enoA*, *gatA*, *hemN*, and *hlfX* genes from geese were identical to those of the patients. Meanwhile, patient 3 had a history of butchering geese. Based on epidemiological survey data, the four patients did not know each other. They lived in two different counties and worked far from one another, which rules out the possibility of transmission among the four patients. However, three out of four family members were tested positive for IgG antibodies against *C. psittaci* by serological testing. Since patient 1 and 4 denied having any history of close contact with live birds/poultry, the infections of their family members could not be ruled out the possibility of human-to-human transmission. According to the molecular evidence and the exposure history of patient 2 and 3, we can speculate that the *C. psittaci*-infected geese might be the potential infectious source of human psittacosis in Lishui city.

Psittacosis, a zoonotic infectious disease, caused by *C. psittaci* transmitted commonly from birds/poultry to human. There have been a few reports about the clustered cases of psittacosis in families or hospitals, indicating the possibility of person-to-person transmission of the disease [15,16]. In the present study, two of the four patients denied having any history of close contact with live birds/poultry, suggesting they might be infected via inhalation of *C. psittaci* organisms in the environment that might be contaminated by the faeces from poultry infected by *C. psittaci*.

In the present study, *C. psittaci* genotype E/B strain was isolated from one of four consecutive psittacosis patients and the *ompA* of *C. psittaci* was amplified from the samples of all the four patients and two geese at the home of patient 2 in Lishui city of China. And the amplified *ompA* of *C. psittaci* were identical to one another in DNA sequences assay, indicating that genotype E/B strain has circulated in the local poultry and the geese infected with *C. psittaci* might be the infectious source of the human psittacosis. The present report is believed to be the first on *C. psittaci* genotype E/B transmission from geese to humans. Two patients denied having any history of close contact with live poultry, suggesting that they might be infected via inhalation of the *C. psittaci* organisms in the environment that might be contaminated by the faeces from the poultry infected with *C. psittaci*, which may raise a public health concern. Therefore, there is an urgent need to strengthen the *C. psittaci* surveillance among poultry and humans for prevention and control the outbreak of psittacosis in the city.

## Supplementary Material

Supplemental Material
