# Supplementary material for: Severe community-acquired pneumonia caused by Chlamydia psittaci genotype E/B strain circulating among geese in Lishui city, Zhejiang province, China
Source: Emerg Microbes Infect. 2022 Nov 10;11(1):2715–23. doi: 10.1080/22221751.2022.2140606 (PMC9661978; doi:10.1080/22221751.2022.2140606)
Supplement: Supplemental Material [file TEMI_A_2140606_SM4723.zip › Supplementary materials.docx]

**Supplemental Methods**

**MinION sequencing**

The whole genome of *C. psittaci* LS strain was sequenced using MinION Nanopore (Oxford Nanopore Technologies, Oxford, UK) method. The library was prepared for MinION Nanopore sequencing using a Genomic DNA ligation kit (SQK-LSK109; Oxford Nanopore Technologies) according to the manufacture’s protocols. DNA libraries were loaded onto FLO-MIN106 flow cells (R9.4.1), and sequenced using the MinION Mk1C for 72 h.

**Illumina sequencing**

The genomic DNA was sheared using sonication device for the construction of short-insert paired-end (PE) libraries. The short-insert (~500 bp) libraries were constructed as described in Illumina library preparation kit. All libraries were sequenced on an Illumina X-TEN platform (San Diego, CA, USA). The raw reads were subsequently trimmed for quality using Trimmomatic [1] (v.0.35) with the parameters “ILLUMINACLIP: TruSeq3-PE.fa:2:30:10 LEADING: 3 TRAILING: 3 SLIDINGWINDOW: 4:15 MINLEN: 36”. The clean data obtained from this process were used for subsequent analysis.

**Genome assembly**

Base calling of the fast5 files was performed using GUPPY (version 1.4.3-1; Oxford Nanopore Technologies). Reads were then BLAST searched against the NCBI nucleotide (nt) database.[2] All long reads related to the genus *LS* strain were mapped to the reference *C. psittaci* WS/RT/E30 genome sequence (GenBank accession number CP003794) using Minialign 0.5.3.[3]To improve the accuracy of our assembly, the whole‑genome Illumina short reads were mapped to the Oxford Nanopore long reads using BWA-MEM and errors were corrected.[4] Gene annotation was performed using Prodigal V2.6.3.[5]

**Core-gene analysis**

Genome sequences of 15 *C. psittaci* strains were downloaded from the NCBI. Snippy [6] was used to finds SNPs to generate a core SNP alignment. The recombinant regions were identified and excluded using the software package Gubbins.[7] Maximum likelihood trees were estimated using IQtree v.1.6.9.[8] The bacterial strains used in this tree are shown in Supplementary Table S3.

The genome of *C. psittaci* LS strain was compared with seven representative strain of A to E, and E/B genotypes to find common genes shared with other genotype, and unique genes that are only present in this new strain. Then the specific genes were annotated by gene ontology (GO) terms and analyzed using WEGO program [9].

**References for supplemental methods**

1. Bolger AM, Lohse M, Usadel B. Trimmomatic: a flexible trimmer for Illumina sequence data. Bioinformatics 2014;30:2114-20.

2. Altschul SF, Gish W, Miller W, Miller W, Lipman DJ. Basic local alignment search tool. J Mol Biol 1990;215:403-410.

3. Suzuki H, Kasahara M. Introducing difference recurrence relations for faster semi-global alignment of long sequences. BMC Bioinformatics 2018;19:45.

4. Li H, Durbin R. Fast and accurate short read alignment with Burrows-Wheeler transform. Bioinformatics 2009;25:1754-60.

5. Hyatt D, Chen GL, Locascio PF, Land ML, Larimer FW, Hauser LJ. [Prodigal: prokaryotic gene recognition and translation initiation site identification.](https://pubmed.ncbi.nlm.nih.gov/20211023/)

BMC Bioinformatics. 2010;11:119.

6. Seemann T (2015) snippy: fast bacterial variant calling from NGS reads https://github.com/tseemann/snippy.

7. Croucher NJ, Page AJ, Connor TR, et al. Rapid phylogenetic analysis of large samples of recombinant bacterial whole genome sequences using Gubbins. Nucleic Acids Res 2015;43:e15.

8. Nguyen LT, Schmidt HA, von Haeseler A, Minh BQ. IQ-TREE: a fast and effective stochastic algorithm for estimating maximum-likelihood phylogenies. Mol Biol Evol 2015;32:268-74.

9. Ye J, Zhang Y, Cui H, et al. WEGO 2.0: a web tool for analyzing and plotting GO annotations, 2018 update. Nucleic Acids Res. 2018;46(W1):W71-W75.
