# Supplementary material for: Severe community-acquired pneumonia caused by Chlamydia psittaci genotype E/B strain circulating among geese in Lishui city, Zhejiang province, China
Source: Emerg Microbes Infect. 2022 Nov 10;11(1):2715–23. doi: 10.1080/22221751.2022.2140606 (PMC9661978; doi:10.1080/22221751.2022.2140606)
Supplement: Supplemental Material [file TEMI_A_2140606_SM4723.zip › Table S1.docx]

Table S1. Samples of collected in this study.

| Types of samples | *No. of samples | Percentage of positive(%) |
| --- | --- | --- |
| BALF | 2/2 | 100 |
| sputum | 2/2 | 100 |
| Food of poultry | 0/11 | 0 |
| Duck feces | 0/7 | 0 |
| Duck Oropharyngeal swabs | 0/6 | 0 |
| Duck feather | 0/1 | 0 |
| Geese feces | 2/4 | 50 |
| Chickens’ feces | 0/4 | 0 |
| Chickens’ Anal swab | 0/8 | 0 |
| Chickens’ Oropharyngeal swabs | 0/3 | 0 |
| Environmental | 0/18 | 0 |
| total | 6/66 | 9.09 |

*No. of positive samples/No. of samples collected
