# Supplementary material for: Severe community-acquired pneumonia caused by Chlamydia psittaci genotype E/B strain circulating among geese in Lishui city, Zhejiang province, China
Source: Emerg Microbes Infect. 2022 Nov 10;11(1):2715–23. doi: 10.1080/22221751.2022.2140606 (PMC9661978; doi:10.1080/22221751.2022.2140606)
Supplement: Supplemental Material [file TEMI_A_2140606_SM4723.zip › Table S2.docx]

Table S2. The titers of IgG antibodies against C. Psittaci of the patients and their family members.

|  | Family 1 | Family 2 | Family 3 | Family 4 |
| --- | --- | --- | --- | --- |
| patient | 1:128 | 1:256 | 1:256 | 1:1024 |
| Family members 1 | 1:32 | 1:128 | 1:256 | NA |
| Family members 2 | 1:64 | NA | NA | NA |
