# Supplementary material for: Severe community-acquired pneumonia caused by Chlamydia psittaci genotype E/B strain circulating among geese in Lishui city, Zhejiang province, China
Source: Emerg Microbes Infect. 2022 Nov 10;11(1):2715–23. doi: 10.1080/22221751.2022.2140606 (PMC9661978; doi:10.1080/22221751.2022.2140606)
Supplement: Supplemental Material [file TEMI_A_2140606_SM4723.zip › Table S3.docx]

Table S3. Genome properties of representative *C. psittaci* genotypes

| Genotypes­ | A | B | C | D | E | F | E/B | E/B |
| --- | --- | --- | --- | --- | --- | --- | --- | --- |
| Strain | 84/55 | CP3 | GR9 | NJ1 | MN | VS225 | WS/RT/E30 | LS |
| NCBI RefSeq | CP003790 | CP003797 | CP003791 | CP003798 | CP003792 | CP003793 | CP003794 | CP098512 |
| Size (bp) | 1,179,551 | 1,175,702 | 1,147,152 | 1,168,986 | 1,175,981 | 1,164,938 | 1,148,342 | 1,157,301 |
| GC (%) | 39.02 | 39.01 | 39.07 | 38.91 | 39.01 | 38.9 | 38.99 | 39.01 |
| Protein | 1,078 | 1,071 | 994 | 998 | 1,010 | 1,061 | 1,005 | 1,094 |
| tRNA | 82 | 85 | 83 | 82 | 81 | 85 | 83 | 87 |
| rRNA | 6 | 6 | 6 | 6 | 6 | 6 | 6 | 6 |
| Other RNA | 3 | 3 | 3 | 3 | 3 | 3 | 3 | 3 |
| Pseudogene | 208 | 183 | 101 | 84 | 99 | 175 | 124 | 247 |
| Total Gene | 1,377 | 1,348 | 1,187 | 1,173 | 1,199 | 1,330 | 1,221 | 1,437 |
