# Supplementary material for: Severe community-acquired pneumonia caused by Chlamydia psittaci genotype E/B strain circulating among geese in Lishui city, Zhejiang province, China
Source: Emerg Microbes Infect. 2022 Nov 10;11(1):2715–23. doi: 10.1080/22221751.2022.2140606 (PMC9661978; doi:10.1080/22221751.2022.2140606)
Supplement: Supplemental Material [file TEMI_A_2140606_SM4723.zip › Table S4.docx]

Table S4: *The C. psittaci* strains were used to construct the phylogeny tree.

| **Strain** | **Assembly accession** | **RefSeq ID** | **Genotype** | **Date** | **Country** | **Host** |
| --- | --- | --- | --- | --- | --- | --- |
| 84/55 | GCF_000298375.2 | CP003790 | A | - | Germany | *Amazona sp.* |
| 02DC15 | GCF_000270425.1 | NC_017292 | A | 2002 | Germany | *Bos taurus* |
| 08DC60 | GCF_000270445.1 | NC_017290 | A | 2008 | Germany | *Homo sapiens* |
| GIMC 2005:CpsCP1 | GCF_002752735.1 | NZ_CP024451 | A | 1971 | Russia: Moscow | *Homo sapiens* |
| 6BC | GCF_000191925.1 | NC_015470 | A | 1983 | USA | Parakeet |
| Ful127 | GCF_003999235.1 | NZ_CP033059 | - | 2016 | Faroe Islands | Fulmars |
| CP3 | GCF_001401455.1 | CP003797 | B | 2015 | USA | *Columba livia* |
| VS225 | GCF_000298455.2 | CP003793 | F | 1991 | USA | Parakeet |
| GR9 | GCF_000298415.1 | NC_018620 | C | 1960 | Germany | *Anas platyrhynchos* |
| Rostinovo-70 | GCF_006385615.1 | NZ_CP041038 | C | 1905 | Russia | *Bos taurus* |
| AMK | GCF_009857155.1 | NZ_CP047319 | C | 2017 | Russia: Volga Region | *Bos taurus* |
| MN | GCF_000298435.2 | NC_018627 | E | 1936 | USA | *Homo sapiens* |
| WS/RT/E30 | GCF_000298475.2 | NC_018622.1 | E/B | 2001 | Germany | *Anas platyrhynchos* |
| WC | GCF_000298515.2 | NC_018624 | WC | 1960 | USA | *Bos taurus* |
